# Realist review of informal carer involvement in the transition of medicines-related care for patients moving from hospital to home

**DOI:** 10.1136/bmjopen-2025-107826

**Published:** 2025-11-13

**Authors:** Charlotte Lucy Richardson, Matthew Cooper, Olivia Atkinson, David Black, Laura Lindsey, Christina Cooper, Hamde Nazar, Geoff Wong, Carmel Hughes

**Affiliations:** 1 National Institute for Health and Care Research Newcastle Patient Safety Research Collaborative, Newcastle University, Newcastle upon Tyne, UK; 2 School of Pharmacy, Newcastle University, Newcastle Upon Tyne, UK; 3 Public Contributor to the School of Pharmacy, Newcastle University, Newcastle upon Tyne, UK; 4 Faculty of Health and Life Sciences, Northumbria University, Newcastle upon Tyne, UK; 5 Nuffield Department of Primary Care Health Sciences, Oxford University, Oxford, UK; 6 Primary Care Research Group, School of Pharmacy, Queen's University Belfast, Belfast, UK

**Keywords:** Caregivers, Hospital to Home Transition, Medicine, Medication Adherence

## Abstract

**Abstract:**

**Objective:**

The aim of this work was to understand carer involvement in transitions of care from hospital to home in relation to medicines management. Specifically, *via* a realist review, to describe how carers provide support, to what extent do they support patients and under what circumstances are carers able to provide support towards patient care in relation to medicines management.

**Design:**

A realist review was conducted in line with a published protocol and as registered *via* PROSPERO (CRD42021262827). An initial programme theory (PT) was developed before searches of three databases, PubMed, CINHAL and EMBASE, were conducted in accordance with eligibility criteria. Data were extracted from eligible studies and synthesised into realist causal explanations in the form of Context-Mechanism-Outcome-Configurations (CMOCs) and the PT was refined. Throughout the review, a patient and PPIE group (n≥5) was involved, meeting five times, to inform the research focus and develop CMOCs and the PT by providing feedback and ensuring they capture the carer experience.

**Results:**

Following title and abstract screening of 4835 papers, the final number of included articles was 208. The evidence synthesis identified 31 CMOCs which were categorised into three themes: (1) continuum of support; (2) understanding the carers’ priorities, role and responsibilities through shared decision-making (SDM) and (3) access to appropriate materials, resources and support information. These themes were formed into an updated PT with accompanying narrative that explained the transition from hospital to home involving carers in medicines management and identified possible areas for future intervention development.

**Conclusion:**

This review provides insights and recommendations on how carers can be better supported when managing medicines when patients are discharged from hospital. Carers need a continuum of support throughout and following the transition. Healthcare professionals can support this by understanding the carer’s priorities, role and responsibilities through SDM during the hospital stay. Consequently, carers can then be offered access to appropriate materials, resources and support information which allows them to provide better care relating to medicines in the long term.

**STRENGTHS AND LIMITATIONS OF THIS STUDY**

- This work reports the findings of an already published protocol (10.1136/bmjopen-2024–091005) using the Realist and Meta-narrative Evidence Syntheses: Evolving Standards to ensure transparency and rigour.
- A diverse patient and public involvement and engagement group that contributed to the lifecycle of the research, with one author having lived experiences as a carer.
- Non-English language articles were included within this review and forwards and backwards translation with bi-lingual speakers ensured accuracy in the extraction of relevant data of these articles.
- Studies relating to non-acute hospital discharge, for example, mental health hospitals, or discharge to other locations other than home were excluded. This literature could have been transferable but was excluded to ensure the volume of literature remained focused and manageable.

## Introduction

 The transition of patients, particularly those who are older and frail with complex medical problems or who take five or more medicines, from hospital to home has been recognised as a risk to patient safety due to issues with communication, incomplete documentation and lack of instruction on medicines at discharge.^1^ These issues can cause medication-related harm (MRH) and negatively impact patients’ quality of life; MRH is estimated to cost the National Health Service (NHS) in the UK £90 million annually.^2^ The risk of MRH appears to be worsened during hospital discharge, with 28% of adults aged above 65 years experiencing MRH within 8 weeks of hospital discharge, resulting in further uptake of NHS services and annually costing the NHS approximately £400 million.^3^

Patients transitioning from hospital to home can experience significant disruption to their usual routines, especially when medicines are changed.^4^ When this disruption is coupled with inadequate explanations about changes to medicines and a lack of care co-ordination, this can exacerbate patient and carer anxiety, create confusion around discharge medicines and further risk patient safety.^5^,^7^ During and following the transition home, many patients rely on informal carers to support medicines use. It is estimated that 5.2 million people in the UK provide informal care.^8^ Carers often take on a significant responsibility of implementing changes to medicines following hospital discharge, which can be increasingly complex.^9^

An informal carer can be defined as:

Anyone who looks after a family member, partner or friend, who needs help because of their illness, frailty, disability, a mental health problem or an addiction and cannot cope without their support. The care they give is unpaid.^10^

This definition is adopted throughout this work and the term ‘carer’ is used synonymously for ‘informal carer’ or ‘caregiver’, and throughout, we refer only to adult carers. Equally, the use of the word home refers to patients who are returning to their preadmission residential location which is community-dwelling and not care home-based or an intermediary service, for example*,* rehabilitation care.

Carers can be involved in a range of medicines management activities, where medicines management is, *“The clinical, cost-effective and safe use of medicines to ensure patients get the maximum benefit from the medicines they need, while at the same time minimising potential harm*”.^11^ From a carer perspective, medicines management has been described to include administration, monitoring and storing of medicines, as well as supporting the patient to understand and follow instructions.^12^ Carer involvement during the transition from hospital to home has been shown to be beneficial to patient outcomes by reducing readmission rates and the risk of MRH by supporting more appropriate use of medicines.^6 13^

Transitioning from hospital to home can be complex, multifaceted, externally influenced and subject to change, so it may not always be clear on how to best involve carers.^14^ One example of available guidance is the nationally commissioned UK Discharge Medicine Service which recommends carer involvement in the discharge process. This service does not specify details on who, how or when this should occur; therefore, it is not clear to healthcare professionals (HCPs) how to best involve carers.^15^ There is a need to examine the complexity associated with carer involvement in medicines use during the transition of patients from hospital to home to better understand what works, for which patient or carer groups, for what benefits and in what context(s).^13^ By applying a realist review methodology, it may be possible to identify improvements for medicines-related patient safety and better support carer involvement during the transition of patients from hospital to home.

### Aim

The aim of this realist review was to understand the role of carers’ involvement in medicines management during the transition between hospital to home. Specifically, how carers provide support, to what extent they support patients and under what circumstances are carers able to provide support for the patients’ medicines management.

The review questions are organised using the Context, Intervention, Mechanism, Outcome framework^16^:

1. Context: what are the contexts in which carer involvement in transitions of care and medicines management support patient outcomes?
  Intervention: what are the details of medicines-related activities that include carers and are provided to aid transition from hospital discharge to homes as described in the literature?
2. Mechanism: what are the context-specific mechanisms which are likely to contribute to reported outcomes?
3. Outcome: what are the outcomes, related to the interaction between the context and the mechanism, achieved by the support from carers in the transition of care relating to medicines management, for people who have been discharged home from hospital?

## Method

The protocol for this realist review was registered with PROSPERO (CRD42021262827) and published.^17^ Ethical approval was not required. This realist review follows the steps outlined by Pawson and is reported in accordance with Realist And MEta-narrative Evidence Syntheses: Evolving Standards (see online supplemental materials 1).^14 18^

Realist approaches acknowledge that interventions are complex and may have different effects for different people, depending on the context of an intervention.^18^ A realist approach (namely realist review) was chosen due to the complex and ever-changing needs and relationships between patients, carers and HCPs to manage medicines during the transition period from hospital to home. This understanding is explained using realist causal explanations that take the form of context (C) +mechanism (M) =outcome (also expressed as Context-Mechanism-Outcome-Configurations; CMOCs). CMOCs are used to explain causation (by a mechanism) of an outcome and link this to specific context—thus providing a context-specific causal explanation for any outcome of interest within a given complex intervention.^19^ Multiple configurations of CMOCs are often needed to explain outcomes within a complex intervention and these are organised into a programme theory (PT).^20^

The study followed a four-step process with an overview detailed below in line with the published protocol^20^; any amendments to the protocol have been highlighted.

### Step 1: development of the initial PT

An initial PT was developed based on relevant literature, previous knowledge of the topic and public involvement. The initial PT depicted multiple key stages in the transition from hospital to home: admission, hospitalisation, discharge, transition and home, along with the possibility of readmission (see online supplemental materials 2).

### Step 2: evidence search

The initial PT defined the parameters for formal data searching. Searches were conducted in January 2024 and were built with support from a specialist librarian and were conducted on PubMed, CINAHL and EMBASE. Citation tracking, sibling papers and snowballing were also used. The search strategy can be viewed in the online supplemental materials of the published protocol.^17^

### Step 3: selection, extracting and organising data

The inclusion and exclusion criteria are listed in table 1. Since the publication of the protocol, evidence relating to paediatric carers was additionally excluded due to the notable difference in experiences and circumstances of this carer group.

**Table 1:** Eligibility criteria

Inclusion	Exclusion
Medicines-related	Not medicines-related
Transition of care (hospital to home, where home relates to a patient’s preadmission residential location)	Other transitions, for example*,* to care homes or respite care
Informal/family carer (adult)	Formal/paid carers
Caring for an adult	Patients who are children
	Carers who are children

Title and abstract and full text screening of data was completed using Rayyan by one reviewer (OA), with a second reviewer (MC) independently screening 10% to ensure consistent application of the criteria. Data from the included articles were coded using NVivo software and a data extraction form which detailed characteristics such as the area of care, method, outcomes and summary of usefulness to this review was used (OA led on coding with MC, all final codes were reviewed by the research team and public contributors).

### Step 4: synthesising evidence and drawing conclusions

Synthesis of data used a realist logic of analysis to develop and then test (confirm, refute or refine) the CMOC.^17^ Data to inform interpretations of the relationship between what was functioning as context or mechanisms for outcomes of interest within the updated PT and CMOCs was sought from both within the same and across included documents. The PT and its constituent CMOCs were discussed regularly within the research team and patient and public involvement and engagement (PPIE) group, who helped focus the findings based on resonance with their lived experience. Disagreement was managed by discussion within the research team to reach consensus and, where relevant additional literature searches.

### Patient and PPIE contribution

PPIE is key in realist reviews to ensure the work remains oriented with lived experience.^21^ PPIE representatives and stakeholders contributed throughout this study and included those with lived experiences of caregiving, patients or representatives of carer organisations across England, for example*,* Durham County Carers. Through five online meetings, the PPIE members (≥5 in each meeting) informed the developing PT by providing their lived experiences relative to the data helping to confirm, refine or refute the CMOCs. Meetings each started with a presentation by a member of the research team followed by guided discussion. The presentation varied depending on the stage of the research but typically included an update on the work and the latest iteration of CMOCs and/or PT. Members were encouraged to provide feedback and make suggestions for improvements based on their experience as a carer. This resulted in iterative development of the CMOCs and PT.

## Results

**Figure 1:**
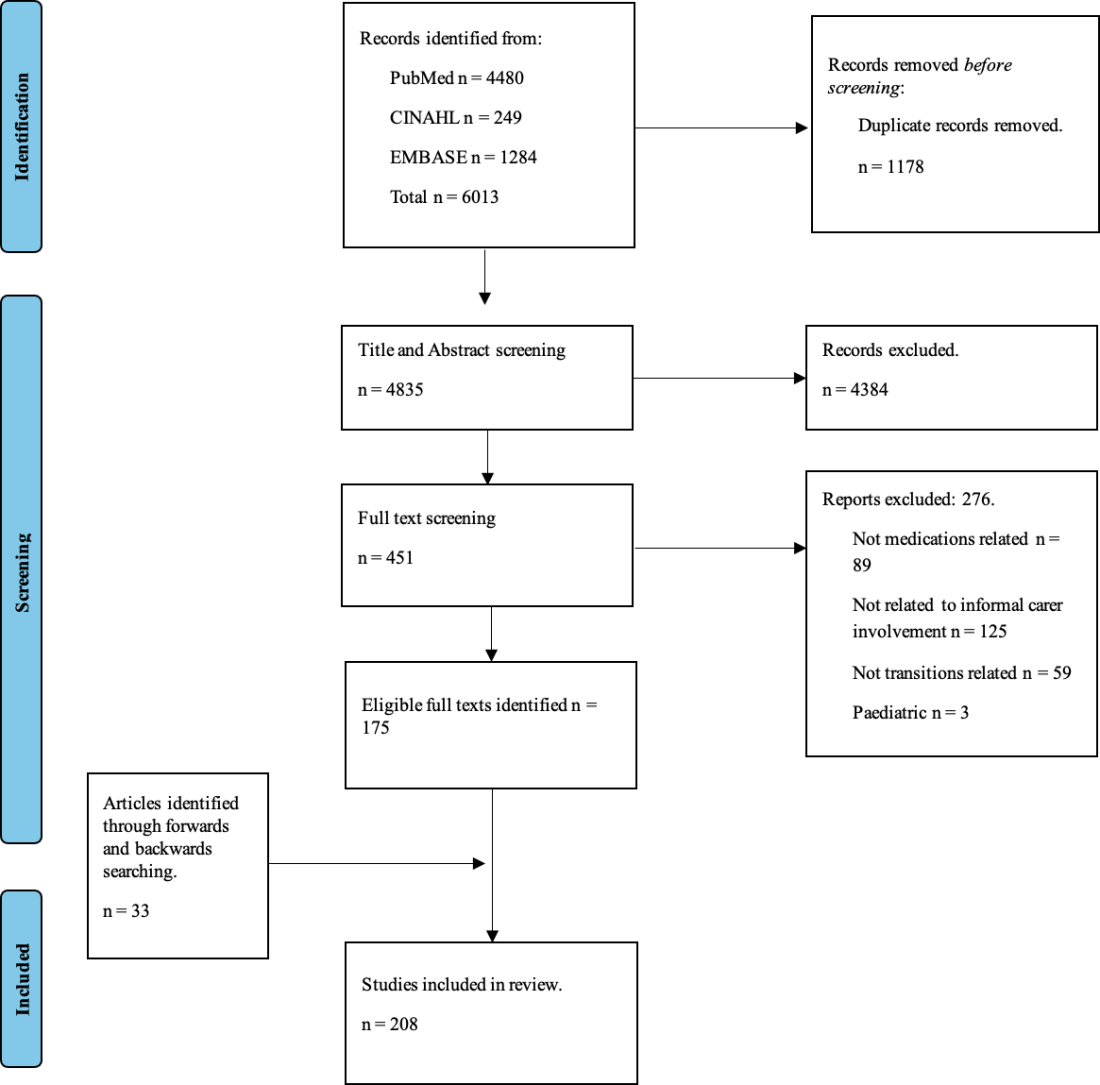
Completed Preferred Reporting Items for Systematic Reviews and Meta-analyses flowchart.

Database searches resulted in 6013 hits. After initial title and abstract screening, removal of duplicates, detailed screening and citation and reference searching, a total of 208 articles were included within the review. The process of identifying, screening and selecting articles is summarised in figure 1 containing the Preferred Reporting Items for Systematic Reviews and Meta-analyses flow diagram.

### Study characteristics

The included articles were published between 1986 and 2023; 144 were full text articles, 44 were conference abstracts and 20 articles were reviews. Just over half were conducted in the USA (n=106; 51%), others were conducted in the UK (n=25; 12%), Canada (n=11; 5%), Australia (n=10; 5%), Switzerland (n=10; 5%) and 26 other countries (n=43; 41.34%) and 1% of articles were conducted in multiple locations (n=3). Most studies used qualitative approaches (n=130, 62.5%), 58 studies used quantitative methods (27.88%) and 20 used mixed methods approaches (9.61%). The majority of studies focused on specific health conditions (n=107; 51.44%) and 71 of the articles did not specify a health condition but had a broader focus (34.13%); stroke (n=24; 11.53%) and heart failure (n=20; 9.6%) were the most common conditions.

### CMOC development

**Figure 2:**
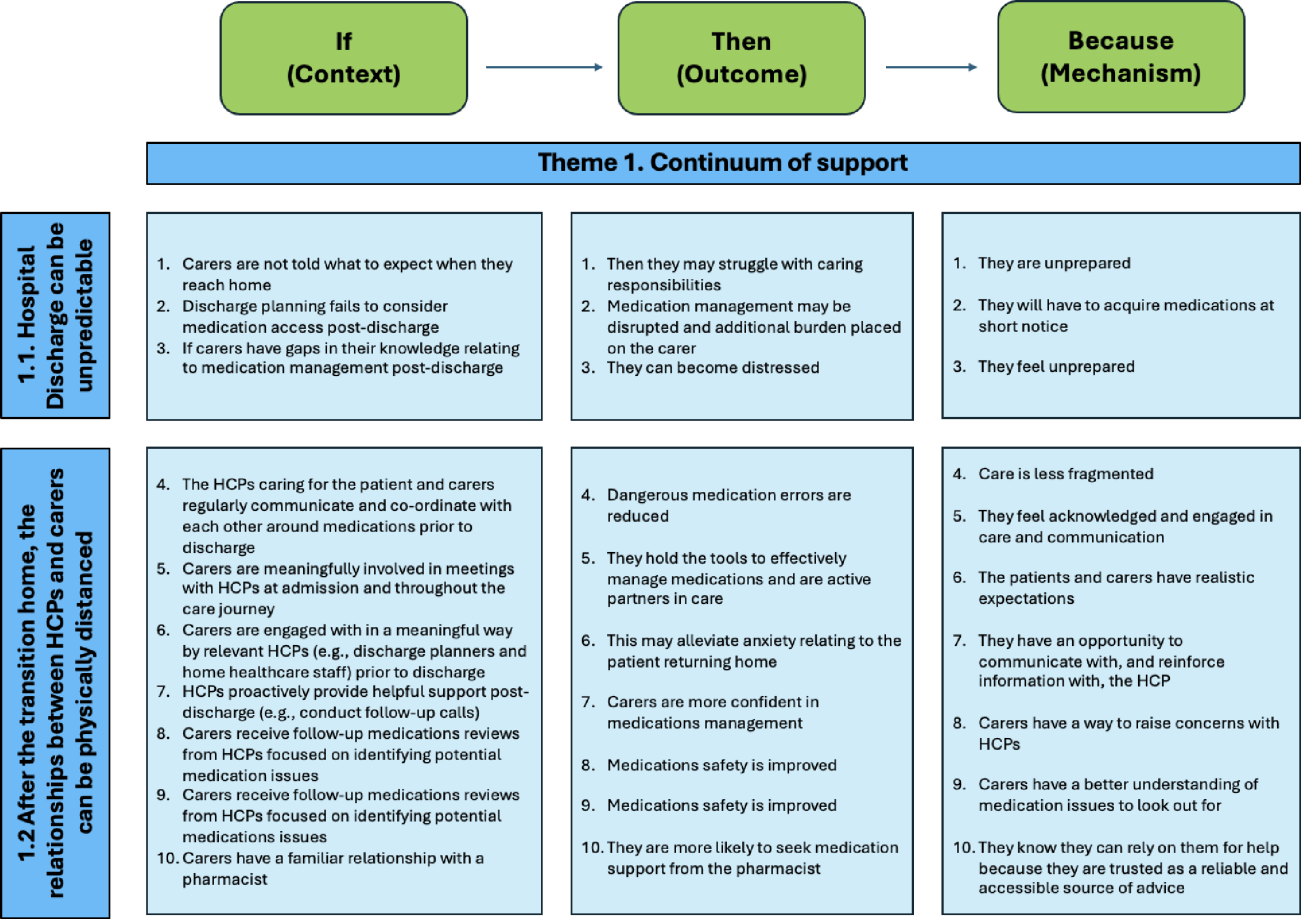
Theme 1 – continuum of support (CMOC 1–10). CMOC, Context-Mechanism-Outcome-Configuration; HCP, healthcare professional.

A total of 31 CMOCs were developed and organised into the three themes that form the updated PT (figure 2), which provides an overarching explanation of the transition from hospital to home involving informal carers in medicines-related interventions. Theme 1: continuum of support, with two subthemes reflecting that hospital discharge can be unpredictable, and after the transition home, the relationship between HCPs and carers can be physically distanced. Theme 2: understanding the carers’ priorities and role and responsibilities through shared decision making (SDM). Theme 3: access to appropriate materials, resources and support information; this theme consists of one subtheme concerning validation on carers’ self-care. Online supplemental material 3 provides a table of all CMOCs and illustrative examples of contributing data sources.

#### Theme 1: continuum of support

**Figure 3:**
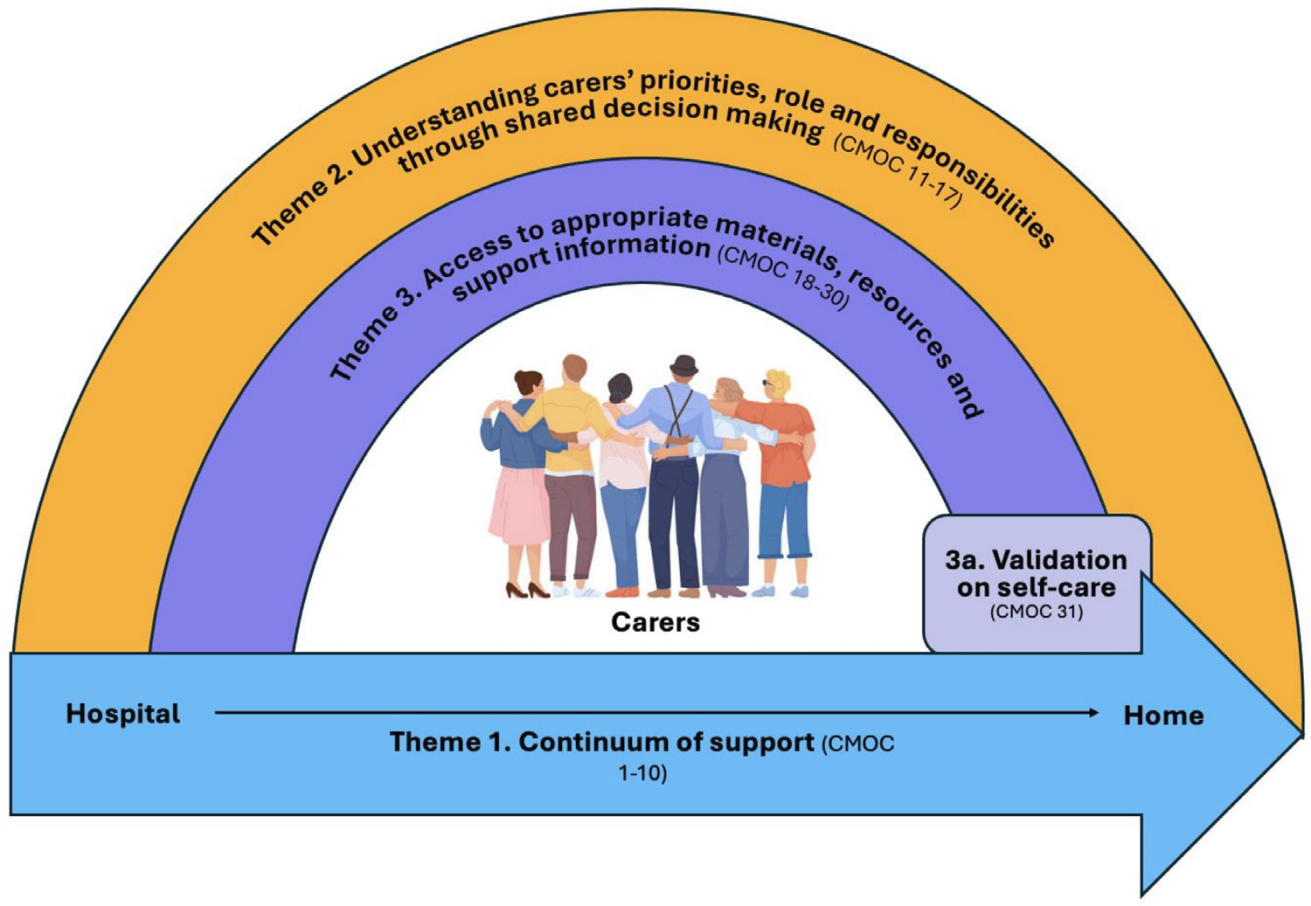
Refined programme theory. CMOC, Context-Mechanism-Outcome-Configuration.

It is important that carers are supported throughout the transition from hospital to home to facilitate a successful transition. This theme captures how the discharge process can be unpredictable, leading to carers’ burden and stress, and how the physically distanced relationship with HCPs after discharge can affect caring ability due to a perceived lack of support by carers (see figure 3).

##### Hospital discharge can be unpredictable

Hospital discharge can be unpredictable for both carers and patients and the unpredictability can also fail to consider the practicalities of medicines supply. The unpredictability can contribute to carer stress and burden if carers do not feel supported (CMOC-1). Examples of issues faced by carers during hospital discharge include discharge occurring at inconvenient times or at late notice to carers, with a small or insufficient supply of medicines and possibly when the pharmacy is closed. This leaves carers trying to obtain medicines at short notice and may contribute to increased carer stress and burden and/or have financial implications (CMOC-2).

During hospital discharge, carers are not always given enough information to care, leaving gaps in their knowledge. This appears to be worsened when discharge is rushed or unexpected to carers and can include carers not being given correct or adequate information relating to medicines changes, drug-drug interactions and possible side effects. Receiving inadequate information means carers go home feeling unprepared, which can negatively impact their well-being, causing distress (CMOC-3).

The unpredictability of discharge is not always controllable due to the inherent nature of a clinical environment, but there are ways to improve the outcome for carers through understanding carers’ roles and responsibilities (theme 2), addressing any perceived distance between carers and HCPs (subtheme 1.2) and through providing appropriate resources for carers (theme 3).

##### After the transition to home, the relationships between HCPs and carers can be physically distanced

After discharge to home, the relationships between HCPs and carers can be physically distanced; therefore, from the perspective of carers, the perception of support can be reduced. This could be addressed and/or anticipated from the point of admission by involving carers in meetings with HCPs throughout the care journey and through the use of SDM. Involvement in SDM can allow carers to develop the skills and confidence to become an active partner in care following the transition home, helping them to feel more acknowledged and engaged (CMOC-5). Involvement in SDM can continue throughout the admission so that once the transition to home has occurred, carers have realistic expectations of both the support that will be offered by HCPs and of their roles as a carer in relation to medicines use (CMOC-6).

After hospital discharge, despite physical distance from the patient and carer, HCPs can provide proactive support such as through follow-up phone calls. This can increase carers’ confidence in their ability to manage medicines as they have an opportunity to communicate with, and receive reinforcement from, HCPs (CMOC-7). Specifically, from a medicines perspective, this has the potential to improve medicines safety. Other ways in which follow-up home support could be used to identify potential medicines safety issues is via home medicines reviews to reduce polypharmacy and medicines discrepancies. These also allow carers to gain necessary information around medicines-related issues to be observant of, which in turn could contribute to minimising medicines-related issues (CMOC-8).

Following hospital discharge, a long-term, familiar and positive relationship with a community pharmacist can be important. When carers trust the pharmacist, they are more likely to seek medicines support from them, encouraging collaboration. Carers know they can rely on pharmacists for help because they are a trusted, reliable and accessible source of medicines related information (CMOC-10).

### Theme 2: understanding the carers’ priorities, role and responsibilities through SDM

**Figure 4:**
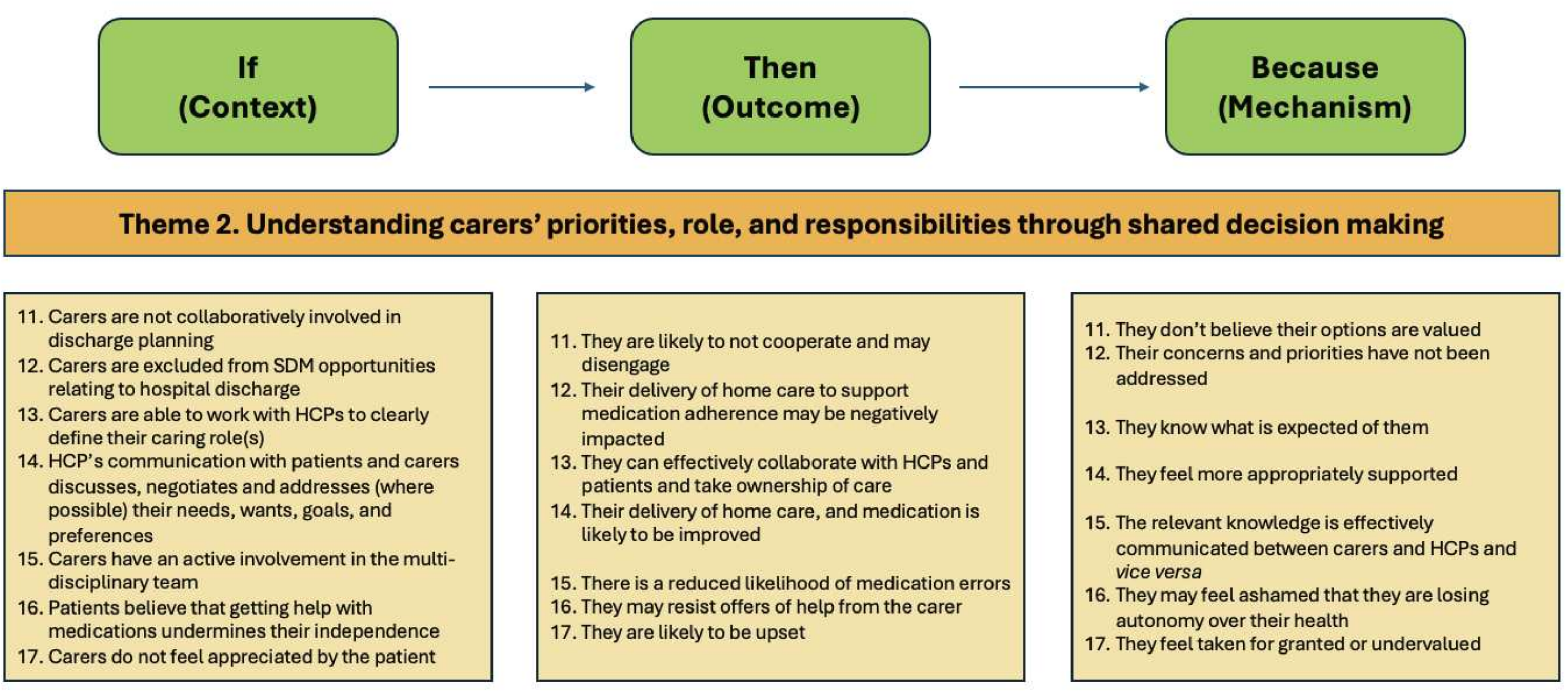
Theme 2 - understanding carers’ priorities, role and responsibilities through shared decision making (CMOC 11-17). CMOC, Context-Mechanism-Outcome-Configuration, HCP, healthcare professional; SDM, shared decision making.

Carers can be supported during their transition from hospital to home when HCPs have a better understanding of the carer’s priorities, role and responsibilities through making use of SDM (figure 4).

Including carers and patients in SDM can help to identify and consider individual carers’ and patients’ needs and priorities. This includes aspects relating to the patient’s and carer’s identity, religion and culture as examples, and can help the patient, carer and HCPs to understand the individual carer’s preferred role and responsibilities.

When carers are not involved in SDM, they do not have an opportunity to raise their concerns and priorities and, as such, carers’ delivery of care at home can be negatively impacted, and they may not feel valued (CMOC-11 and CMOC-12). Conversely, by working with HCPs, carers can define their roles and responsibilities; then, once the patient has transitioned home, carers can take ownership of care as they are aware of what is expected of them, and this is built on their own preferences (CMOC-13).

When HCPs provide person-focused care to both the patient and carer, which includes aspects related to cultural nuances, it allows their personal needs, wants, goals and preferences to be met. Thus, supporting carers in being seen and heard may consequently increase medicines adherence and reduce medicines errors (CMOC-14). The potential reduction in medicines errors could occur when carers are collaboratively involved in discharge planning through SDM, and relevant knowledge around medicines is additionally transferred to carers (CMOC-15).

### Theme 3: access to appropriate materials, resources and support information

**Figure 5:**
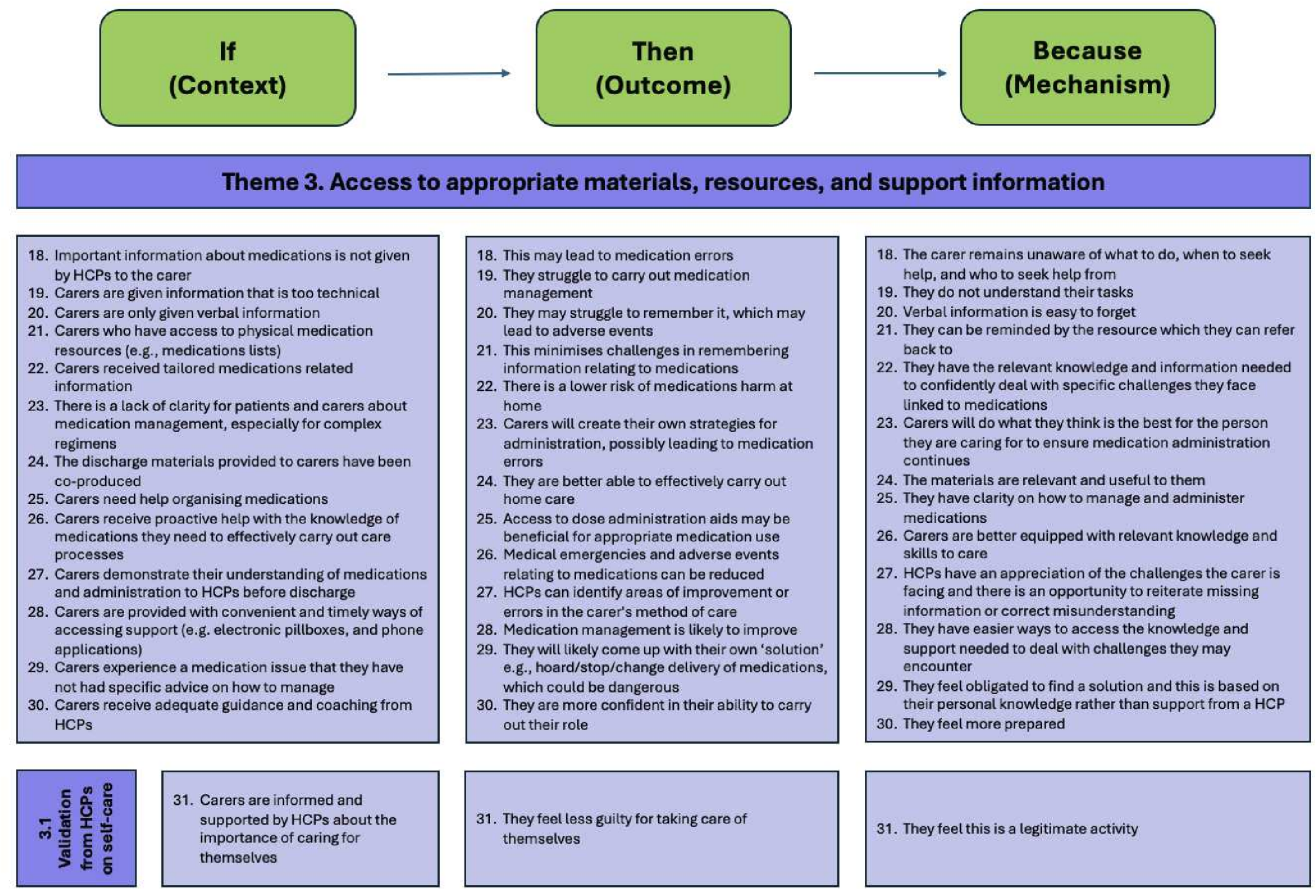
Theme 3: access to appropriate materials, resources and support information (CMOC 18-30). HCP, healthcare professional.

Practically, carers can be supported in several ways by HCPs, but it is crucial that they have access not only to (i) appropriate support and information but also that (ii) they need to receive validation from HCPs as to the need to look after themselves as an important part of their role (figure 5).

If support relating to medicines is not offered to carers, medicines errors can occur because carers are unaware of what to do, when to seek help and who to seek help from (CMOC-18). HCPs should provide carers with important information about the patient’s medicines in advance of discharge; this includes giving the information to the right carer. If not, this may lead to medicines errors as the carer remains unaware of what to do, when to seek help or who to seek help from. This lack of information means that they are unprepared to recognise symptoms, know how to address them or who to seek help from. As an example, when carers are given information at discharge, for example*,* educational resources, the information may be too technical, such as being too long, dense, irrelevant to the individual carer’s needs and full of jargon. Consequently, carers may still struggle to carry out medicines management (CMOC-19).

Patients and carers are sometimes only provided with verbal information on medicines; however, verbal information is easy to forget, especially during the unpredictable period of hospital discharge which can be stressful (CMOC-20). It would be beneficial to provide carers with physical resources to minimise challenges in remembering information relating to medicines, and carers can refer back to them as needed (CMOC-21). Additionally, written information that is tailored to the patient and/or carer helps them to have the relevant knowledge and information needed to confidently deal with the specific challenges linked to medicines they are likely to face (CMOC-22). HCPs may not know what an individual carer’s abilities or requirements are but if materials are shared based on the individual carer’s previously agreed role and responsibilities (theme 2) and the materials are tailored to carers or patients, for example*,* through previous co-design, this gives carers the relevant knowledge and information needed to confidently deal with likely medicines challenges (CMOC-24). When carers do not have access to adequate resources and they do not have clarity on medicines management, especially for complex regimens, then carers create their own strategies for administration, which may lead to medicines errors. Carers often do this in the best interests of the person they are caring for to ensure medicines administration continues, but this comes with risk due to a lack of support from HCPs (CMOC-23).

After the transition home, when carers begin managing medicines, having timely ways of accessing support convenient for them is important. Some of this can be proactively addressed during the admission *via* coaching by HCPs. The obvious outcome of this is that carers are better equipped with the relevant knowledge and skills and so medical emergencies and adverse events relating to medicines can be reduced, but equally the outcomes of offering proactive support around medicines knowledge is that carers develop confidence in their abilities (CMOC-27 and CMOC-30).

#### Receiving validation from HCPs on self-care

Caring roles can be burdensome on carers, leading them to feel sad, doubt their caring abilities and feel guilty for having their own needs. Carers can be informed of, and supported by HCPs, as to the importance of caring for themselves. This could be through creating self-care plans, receiving information on support services, encouraging carers to ask their social support network for help to prevent burnout and framing the discussion from the perspective that staying healthy means they provide better care. These methods can make carers feel less guilty for self-care because it is validated as a legitimate activity by a HCP (CMOC-31).

## Discussion

This review provides an overall explanation of the barriers and opportunities across the transition from hospital to home through 31 CMOCs across three themes: (1) continuum of support, (2) understanding the carers’ priorities, role and responsibilities through SDM and (3) access to appropriate materials, resources and support information.

Continuum of support highlights the need for support for carers across the transition from hospital to home. Unpredictable discharge times, locations and medicines availability can increase carer burden, especially during weekend discharges when organisation and time pressures on HCPs can be worsened.^22^ Previous research with patients, carers and HCPs highlights that poorly prepared or short notice discharges cause stress for carers, especially when discharge occurs on weekends and evenings when there is more limited medicines availability, which was also highlighted in our review.^23^ While the discharge process can be stressful for carers, this can be mitigated by providing opportunities for carers to engage as active partners in care and build relationships with HCPs. Engaging carers can alleviate carer anxiety, enhance communication and manage expectations of all parties, whereas exclusion of carers from discharge planning meetings can leave carers feeling undervalued and may lead to medicines non-adherence.^24^

Multiple points of contact for carers across the transition offer opportunities both for medicines-related advice and broader carer support, both thought to improve carers’ ability to provide homecare. In a study using a follow-up telephone call service, carers’ needs changed over time—they addressed patient issues and their own education of the clinical condition first, before addressing their emotional and physical health needs, suggesting a need for several points of contact.^25^ Community pharmacies could offer a place to foster long-term relationships with carers whereby communication between pharmacists and carers enhances medicines management, carer understanding of their role and, indirectly, carer health and well-being.^26 27^

The second theme of the PT focuses on the benefits of SDM to better understand carers’ preferred roles and responsibility and their priorities once the patient transitions home. Prior research notes that carers’ own fears and desires may influence their assumptions about treatments and thus should be considered by HCPs.^28^ Involving carers in SDM and giving them opportunities to voice concerns allows for more informed decision making, better tailoring of support and resources offered and thus better homecare following the transition home. However, HCPs should be conscious of how this may affect the patients’ feeling of autonomy and control.^29^

The PT highlights the need to provide carers with appropriate materials, resources and support information. This is already recognised as important for managing medicines at home.^30 31^ HCPs can determine carers’ understanding of roles and tasks by getting carers to demonstrate or ‘teach back’ before discharge, providing an opportunity to identify and correct errors.^32^,^35^ Tailored resources, feedback and support can be offered as physical materials, verbally or combined but, regardless, should be clear and accessible. For example, verbal-only information risks limited recall,^22^ and language (written or verbal) needs to be free from jargon and account for the carer’s health literacy.^22 36^ The suitability of materials could be achieved through co-production with carers, which has been shown to foster collaboration and trust between HCPs and carers.^37^,^39^ Given the high burden on carers, which is known to contribute to anxiety and depression,^40^ it is also important to give carers access to resources addressing the importance of self-care to legitimise the activity.^41 42^

### Strengths and limitations

A strength of this study was the diverse PPIE group that contributed to the lifecycle of the research; DB contributed as a co-researcher as a person with lived experiences as a carer. Representatives of carer organisations, patients and carers contributed throughout, informing the PT, validating CMOC development and suggesting dissemination techniques. A further strength was the inclusion of non-English language articles (n=2). Forwards and backwards translation with bi-lingual speakers ensured accuracy in the extraction of relevant data of these articles.

To ensure a focused search strategy, studies relating to non-acute hospital discharge, for example, mental health hospitals or discharge to other locations other than home were excluded. This literature could have been transferable but was excluded to ensure the volume of literature remained focused and manageable.

### Further research and implications

There is a need for increased recognition of carers as essential partners in post-hospital discharge care both on the level of individual HCPs in practice and in wider policy and discharge protocols. This could facilitate more structured carer involvement in SDM which balances the carer role with carer burden and needs. Future work could consider the development of an intervention to address areas of the PT, particularly around involving carers in SDM and providing carers with appropriate and tailored resources; this may include the use of co-design. Another area of focus may be to explore the experiences of carers being able to consult the same HCPs on an ongoing basis, including community pharmacists.

## Conclusion

This review provides insights and recommendations to increase patient safety related to medicines use following a transition from hospital to home by better involving and supporting carers. Carers need a continuum of support, across the hospital stay and transition, and this starts from HCPs understanding the individual carers’ priorities, role and responsibilities through SDM. As a result, carers can then be offered access to appropriate materials, resources and support information which allows them to provide better support in the long term as well as looking after themselves.

## Supplementary material

10.1136/bmjopen-2025-107826 online supplemental file 1

## Data Availability

Data are available upon reasonable request.
