# Supplementary material for: Realist review of informal carer involvement in the transition of medicines-related care for patients moving from hospital to home
Source: BMJ Open. 2025 Nov 13;15(11):e107826. doi: 10.1136/bmjopen-2025-107826 (PMC12625932; doi:10.1136/bmjopen-2025-107826)
Supplement: online supplemental file 1 [file bmjopen-15-11-s001.docx]

1. **RAMESES Guidelines**

| **Guideline Item** | **Page number** |
| --- | --- |
| **Title** |  |
| 1. In the title, identify the document as a realist synthesis or review. | 1 |
| **Abstract** |  |
| 1. While acknowledging publication requirements and house style, abstracts should ideally contain brief details of: the study's background, review question or objectives; search strategy; methods of selection, appraisal, analysis and synthesis of sources; main results; and implications for practice. | 2 |
| **Introduction** |  |
| 1. Rationale for review: Explain why the review is needed and what it is likely to contribute to existing understanding of the topic area. 2. Objectives and focus of review: State the objective(s) of the review and/or the review question(s). Define and provide a rationale for the focus of the review. | 4  5 |
| **Methods** |  |
| 1. Changes in the review process: Any changes made to the review process that was initially planned should be briefly described and justified. 2. Rationale for using realist synthesis: Explain why realist synthesis was considered the most appropriate method to use. 3. Scoping the literature: Describe and justify the initial process of exploratory scoping of the literature. 4. Searching processes: While considering specific requirements of the journal or other publication outlet, state and provide a rationale for how the iterative searching was done. Provide details on all the sources accessed for information in the review. Where searching in electronic databases has taken place, the details should include, for example, name of database, search terms, dates of coverage and date last searched. If individuals familiar with the relevant literature and/or topic area were contacted, indicate how they were identified and selected. 5. Selection and appraisal of documents: Explain how judgements were made about including and excluding data from documents, and justify these. 6. Data extraction: Describe and explain which data or information were extracted from the included documents and justify this selection. 7. Analysis and synthesis processes: Describe the analysis and synthesis processes in detail. This section should include information on the constructs analysed and describe the analytic process. | 8  6  6  6  6  8  8 |
| **Results** |  |
| 1. Document flow diagram: Provide details on the number of documents assessed for eligibility and included in the review with reasons for exclusion at each stage as well as an indication of their source of origin (for example, from searching databases, reference lists and so on). You may consider using the example templates (which are likely to need modification to suit the data) that are provided. 2. Document characteristics: Provide information on the characteristics of the documents included in the review. 3. Main findings: Present the key findings with a specific focus on theory building and testing. | 10  9  10 |
| **Discussion** |  |
| 1. Summary of findings: Summarize the main findings, taking into account the review's objective(s), research question(s), focus and intended audience(s). 2. Strengths, limitations and future research directions: Discuss both the strengths of the review and its limitations. These should include (but need not be restricted to) (a) consideration of all the steps in the review process and (b) comment on the overall strength of evidence supporting the explanatory insights which emerged. The limitations identified may point to areas where further work is needed. 3. Comparison with existing literature: Where applicable, compare and contrast the review's findings with the existing literature (for example, other reviews) on the same topic. 4. Conclusion and recommendations: List the main implications of the findings and place these in the context of other relevant literature. If appropriate, offer recommendations for policy and practice. 5. Funding: Provide details of funding source (if any) for the review, the role played by the funder (if any) and any conflicts of interests of the reviewers. | 19  20  19  21  22 |

**2. Initial programme theory before refinement**


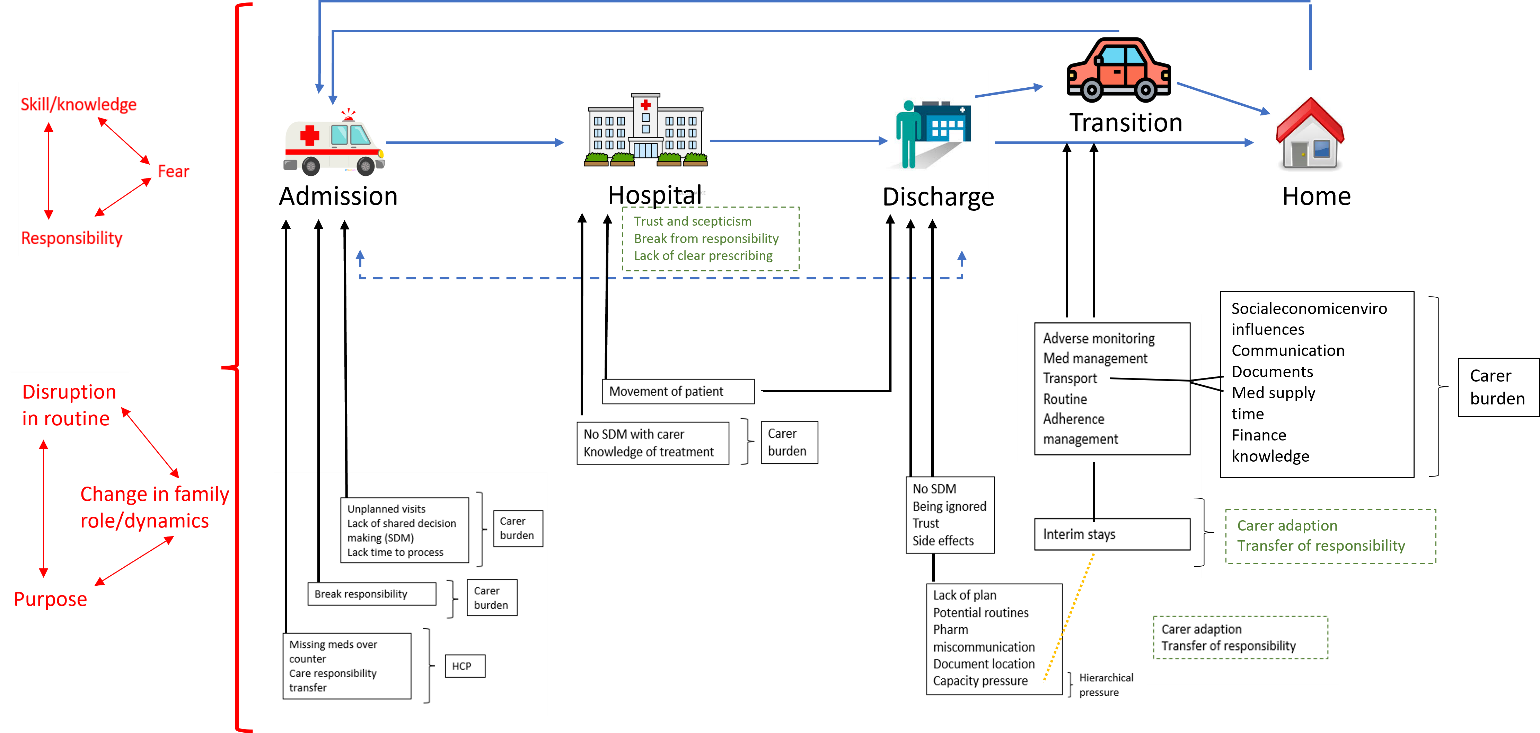


**3. CMOCs and illustrative examples of contributing data source(s)**

| **CMOC** | **Illustrative Data Source(s)** |
| --- | --- |
| **Theme 1: Continuum of support** | |
| **1.1 Hospital discharge can be unpredictable** | |
| **1. IF** Carers are not told what to expect when they reach home  **THEN** they may struggle with caring responsibilities  **BECAUSE** They are unprepared | - **Knight DA, Thompson D, Mathie E, Dickinson A.** 'Seamless care? Just a list would have helped!' Older people and their carer's experiences of support with medication on discharge home from hospital. Health Expect. 2013 Sep;16(3):277-91. doi: 10.1111/j.1369-7625.2011.00714.x. Epub 2011 Aug 12. PMID: 21838834; PMCID: PMC5060666. - **Saunders S, Weiss ME, Meaney C, et al.** Examining the course of transitions from hospital to home-based palliative care: a mixed methods study. Palliat Med. 2021;35(8):1590-1601. doi:10.1177/02692163211023682 |
| **2. IF** Discharge planning fails to consider medication access post-discharge  **THEN** Medication management may be disrupted and additional burden placed on the carer  **BECAUSE** They will have to acquire medications at short notice | - **Kiran T, Wells D, Okrainec K, Kennedy C, Devotta K, Mabaya G, Phillips L, Lang A, O'Campo P.** Patient and caregiver experience in the transition from hospital to home - brainstorming results from group concept mapping: a patient-oriented study. CMAJ Open. 2020 Mar 2;8(1):E121-E133. doi: 10.9778/cmajo.20190009. PMID: 32127383; PMCID: PMC7055492. - **Sawan MJ, Jeon YH, Bond C, Hilmer SN, Chen TF, Wennekers D, Gnjidic D.** Caregivers' experiences of medication management advice for people living with dementia at discharge. J Eval Clin Pract. 2021 Dec;27(6):1252-1261. doi: 10.1111/jep.13551. Epub 2021 Feb 15. PMID: 33586327. - **Weaver FM, Perloff L, Waters T.** Patients' and caregivers' transition from hospital to home: needs and recommendations. Home Health Care Serv Q. 1999;17(3):27-48. doi:10.1300/J027v17n03_03 |
| **3. IF** carers have gaps in their knowledge relating to medication management post-discharge  **THEN** They can become distressed  **BECAUSE** They feel unprepared | - **Agarwal KS, Bhimaraj A, Xu J, Bionat S, Pudlo M, Miranda D, Campbell C, Taffet GE.** Decreasing Heart Failure Readmissions Among Older Patients With Cognitive Impairment by Engaging Caregivers. J Cardiovasc Nurs. 2020 May/Jun;35(3):253-261. doi: 10.1097/JCN.0000000000000670. PMID: 32221145. - **Harrop E, Byrne A, Nelson A.** “It’s alright to ask for help”: findings from a qualitative study exploring the information and support needs of family carers at the end of life. BMC Palliat Care. 2014;13:22. doi:10.1186/1472-684X-13-22 - **Sawan MJ, Jeon YH, Bond C, Hilmer SN, Chen TF, Wennekers D, Gnjidic D.** Caregivers' experiences of medication management advice for people living with dementia at discharge. J Eval Clin Pract. 2021 Dec;27(6):1252-1261. doi: 10.1111/jep.13551. Epub 2021 Feb 15. PMID: 33586327. |
| **1.2 After the transition to home the relationships between HCPs and carers can be physically distanced** | |
| **4. IF** The HCPs caring for the patient and carers regularly communicate and co-ordinate with each other around medications prior to discharge  **THEN** Dangerous medication errors are reduced  **BECAUSE** Care is less fragmented | - **Applebaum AJ, Sannes TS.** The importance of honoring family caregiver burden: challenges in mental health care delivery. J Clin Psychol Med Settings. 2025 Jun;32(2):193–201. doi: 10.1007/s10880-024-10051-3. PMID: 39397232. - **Maham N, Koronkowski MJ, Kaur T**. Deprescribing: why does it have to be so hard? A case of irrational polypharmacy. J Am Geriatr Soc. 2020;68(S1):S17. doi:10.1111/jgs.16381 - **Meyer-Massetti C, Meier CR, Guglielmo BJ.** The scope of drug-related problems in the home care setting. Int J Clin Pharm. 2018 Apr;40(2):325-334. doi: 10.1007/s11096-017-0581-9. Epub 2018 Jan 11. PMID: 29322475. |
| **5. IF** Carers are meaningfully involved in meetings with HCPs at admission and throughout the care journey  **THEN** They hold the tools to effectively manage medications and are active partners in care  **BECAUSE** They feel acknowledged and engaged in care and communication | - **Kiran T, Wells D, Okrainec K, Kennedy C, Devotta K, Mabaya G, Phillips L, Lang A, O'Campo P.** Patient and caregiver experience in the transition from hospital to home - brainstorming results from group concept mapping: a patient-oriented study. CMAJ Open. 2020 Mar 2;8(1):E121-E133. doi: 10.9778/cmajo.20190009. PMID: 32127383; PMCID: PMC7055492. - **Sawan MJ, Jeon YH, Bond C, Hilmer SN, Chen TF, Wennekers D, Gnjidic D.** Caregivers' experiences of medication management advice for people living with dementia at discharge. J Eval Clin Pract. 2021 Dec;27(6):1252-1261. doi: 10.1111/jep.13551. Epub 2021 Feb 15. PMID: 33586327. - **Tomlinson J, Silcock J, Smith H, Karban K, Fylan B.** Post-discharge medicines management: the experiences, perceptions and roles of older people and their family carers. Health Expect. 2020 Dec;23(6):1603-1613. doi: 10.1111/hex.13145. Epub 2020 Oct 16. PMID: 33063445; PMCID: PMC7752204. |
| **6. IF** Carers are engaged within a meaningful way by relevant HCPs (e.g., discharge planners and home healthcare staff) prior to discharge  **THEN** This may alleviate anxiety relating to the patient returning home  **BECAUSE** The patients and carers have realistic expectations | - **Weaver FM, Perloff L, Waters T.** Patients' and caregivers' transition from hospital to home: needs and recommendations. Home Health Care Serv Q. 1999;17(3):27-48. doi:10.1300/J027v17n03_03 |
| **7. IF** HCPs proactively provide helpful support post-discharge (e.g., conduct follow-up calls)  **THEN** Carers are more confident in medications management  **BECAUSE** They have an opportunity to communicate with, and reinforce information with, the HCP | - **Bushnell C, Reynolds P. Systematic stroke prevention.** Continuum (Minneap Minn). 2011 Dec;17(6 2ndary Stroke Prevention):1318-34. doi: 10.1212/01.CON.0000410038.72594.4a. PMID: 22810033. - **Cornetta K, Nyariki S, Manji I, et al.** Telehospice for cancer patients discharged from a tertiary care hospital in Western Kenya: a feasibility study. J Pain Symptom Manage. 2023 May;65(5):378–387. doi:10.1016/j.jpainsymman.2023.01.027 - **Dreyer T.** Care transitions: best practices and evidence‑based programs. Home Healthc Nurse. 2014;32(5):309‑316. doi:10.1097/NHH.0000000000000069 |
| **8. IF** Carers receive follow-up medications reviews from HCPs focused on identifying potential medication issues  **THEN** Medications safety is improved  **BECAUSE** Carers have a way to raise concerns with HCPs | - **Callister C, Jones J, Schroeder S, Breathett K, Dollar B, Sanghvi UJ, Harnke B, Lum HD, Jones CD.** Caregiver Experiences of Care Coordination for Recently Discharged Patients: A Qualitative Meta synthesis. West J Nurs Res. 2020 Aug;42(8):649-659. doi: 10.1177/0193945919880183. - **Kable A, Pond D, Baker A, Turner A, Levi C.** Evaluation of discharge documentation after hospitalization for stroke patients discharged home in Australia: A cross-sectional, pilot study. Nurs Health Sci. 2018 Mar;20(1):24-30. doi: 10.1111/nhs.12368. - **Schubert CC, Perkins AJ, Myers LJ, Damush TM, Penney LS, Zhang Y, Schwartzkopf AL, Preddie AK, Riley S, Menen T, Bravata DM.** Effectiveness of the VA-Geriatric Resources for Assessment and Care of Elders (VA-GRACE) program: An observational cohort study. J Am Geriatr Soc. 2022 Dec;70(12):3598-3609. doi: 10.1111/jgs.18013. - **Sawan MJ, Jeon YH, Bond C, Hilmer SN, Chen TF, Wennekers D, Gnjidic D.** Caregivers' experiences of medication management advice for people living with dementia at discharge. J Eval Clin Pract. 2021 Dec;27(6):1252-1261. doi: 10.1111/jep.13551. Epub 2021 Feb 15. PMID: 33586327. - **Tomlinson J, Silcock J, Smith H, Karban K, Fylan B.** Post-discharge medicines management: the experiences, perceptions and roles of older people and their family carers. Health Expect. 2020 Dec;23(6):1603-1613. doi: 10.1111/hex.13145. Epub 2020 Oct 16. PMID: 33063445; PMCID: PMC7752204. |
| **9. IF** Carers receive follow-up medications reviews from HCPs focused on identifying potential medications issues  **THEN** Medications safety is improved  **BECAUSE** Carers have a better understanding of medication issues to look out for | - **Andrade LM, Costa MFM, Caetano JÁ, Soares E, Beserra EP.** A problemática do cuidador familiar do portador de acidente vascular cerebral. Rev Esc Enferm USP. 2009;43(1):37-43. doi:10.1590/S0080-62342009000100005 - **Bruce R, Murdoch W, Kable A, Palazzi K, Hullick C, Pond D, Oldmeadow C, Searles A, Fullerton A, Fraser S, Ling R, Attia J.** Evaluation of Carer Strain and Carer Coping with Medications for People with Dementia after Discharge: Results from the SMS Dementia Study. Healthcare (Basel). 2020 Jul 31;8(3):248. doi: 10.3390/healthcare8030248. - **Perloe M, Keberly ML, Mims AD.** The value of a coach to improve hospital discharges. J Am Geriatr Soc. 2011;59(Suppl 1):S1-S232. doi:10.1111/j.1532-5415.2011.03416.x - **Schubert CC, Perkins AJ, Myers LJ, Damush TM, Penney LS, Zhang Y, Schwartzkopf AL, Preddie AK, Riley S, Menen T, Bravata DM.** Effectiveness of the VA-Geriatric Resources for Assessment and Care of Elders (VA-GRACE) program: An observational cohort study. J Am Geriatr Soc. 2022 Dec;70(12):3598-3609. doi: 10.1111/jgs.18013. - **Weaver FM, Perloff L, Waters T.** Patients' and caregivers' transition from hospital to home: needs and recommendations. Home Health Care Serv Q. 1999;17(3):27-48. doi:10.1300/J027v17n03_03 |
| **10. IF** Carers have a familiar relationship with a pharmacist  **THEN** They are more likely to seek medication support from the pharmacist  **BECAUSE** They know they can rely on them for help because they are trusted as a reliable and accessible source of advice | - **Collier A, Balmer D, Dai L, Hikaka J, Bloomfield K, Boyd M.** Older people, medication safety, and the role of the community pharmacist: a longitudinal ethnographic study. J Pharm Pract Res. 2023;53:18-25. doi:10.1002/jppr.1839 - **Coleman EA, Roman SP.** Family caregivers' experiences during transitions out of hospital. J Healthc Qual. 2015 Jan-Feb;37(1):12-21. doi: 10.1097/01.JHQ.0000460117.83437.b3. - **Schulte N, Ruisinger JF, Prohaska ES, Steele KM, Melton BL.** Comprehensive medication review recipients' opinions, actions, and information recall. J Am Pharm Assoc (2003). 2017 May-Jun;57(3):407-411. doi: 10.1016/j.japh.2017.01.018. - **Tomlinson J, Silcock J, Smith H, Karban K, Fylan B.** Post-discharge medicines management: the experiences, perceptions and roles of older people and their family carers. Health Expect. 2020 Dec;23(6):1603-1613. doi: 10.1111/hex.13145. Epub 2020 Oct 16. PMID: 33063445; PMCID: PMC7752204. |
| **Theme 2: Understanding the carers’ priorities, role and responsibilities through shared decision making** | |
| **11. IF** Carers are not collaboratively involved in discharge planning  **THEN** They are likely to not cooperate and may disengage  **BECAUSE** They don’t believe their options are valued | - **Sawan MJ, Jeon YH, Bond C, Hilmer SN, Chen TF, Wennekers D, Gnjidic D.** Caregivers' experiences of medication management advice for people living with dementia at discharge. J Eval Clin Pract. 2021 Dec;27(6):1252-1261. doi: 10.1111/jep.13551. Epub 2021 Feb 15. PMID: 33586327. |
| **12. IF** Carers are excluded from Shared decision making (SDM) opportunities relating to hospital discharge  **THEN** Their delivery of home care to support medication adherence may be negatively impacted  **BECAUSE** Their concerns and priorities have not been addressed | - **Ferguson CC, Inglis SC, Newton PJ, Middleton S, Macdonald PS, Davidson PM.** Multimorbidity, self-care and frailty: important considerations in anticoagulation in heart failure with atrial fibrillation. Eur J Heart Fail. 2015;17(Suppl 1):5-441. - **Kiran T, Wells D, Okrainec K, Kennedy C, Devotta K, Mabaya G, Phillips L, Lang A, O'Campo P.** Patient and caregiver experience in the transition from hospital to home - brainstorming results from group concept mapping: a patient-oriented study. CMAJ Open. 2020 Mar 2;8(1):E121-E133. doi: 10.9778/cmajo.20190009. PMID: 32127383; PMCID: PMC7055492. - **Pereira F, Bieri M, Del Rio Carral M, Martins MM, Verloo H.** Collaborative medication management for older adults after hospital discharge: a qualitative descriptive study. BMC Nurs. 2022 Oct 24;21(1):284. doi: 10.1186/s12912-022-01061-3. |
| **13. IF** Carers are able to work with HCPs to clearly define their caring role(s)  **THEN** They can effectively collaborate with HCPs and patients and take ownership of care  **BECAUSE** They know what is expected of them | - **Bodenheimer T, Berry-Millett R.** Care management of patients with complex health care needs. Synthesis Project Research Synthesis Report No. 19. Robert Wood Johnson Foundation; December 2009. - **Dolu İ, Naharcı Mİ, Logan PA, Paal P, Vaismoradi M.** A qualitative study of older patients’ and family caregivers’ perspectives of transitional care from hospital to home. Res Theory Nurs Pract. 2021;():. doi:10.1891/RTNP-D-20-00067 - **Greyson S, Keita M, Sharma R, Samus Q, Arbaje AI.** Cultural perceptions of medication management during hospital-to-home transitions of older Latino adults living with dementia. Alzheimers Dement. 2021;17(Suppl):e051181. doi:10.1002/alz.051181 - **Naylor MD, Shaid EC, Carpenter D, Gass B, Levine C, Li J, Malley A, McCauley K, Nguyen HQ, Watson H, Brock J, Mittman B, Jack B, Mitchell S, Callicoatte B, Schall J, Williams MV.** Components of Comprehensive and Effective Transitional Care. J Am Geriatr Soc. 2017 Jun;65(6):1119-1125. doi: 10.1111/jgs.14782. - **White CL, Brady TL, Saucedo LL, Motz D, Sharp J, Birnbaum LA.** Towards a better understanding of readmissions after stroke: partnering with stroke survivors and caregivers. J Clin Nurs. 2015 Apr;24(7-8):1091-100. doi: 10.1111/jocn.12739. |
| **14. IF** HCP’s communication with patients and carers discusses, negotiates and addresses (where possible) their needs, wants, goals, and preferences  **THEN** Their delivery of home care, and medication is likely to be improved  **BECAUSE** They feel more appropriately supported | - **Callister C, Jones J, Schroeder S, Breathett K, Dollar B, Sanghvi UJ, Harnke B, Lum HD, Jones CD.** Caregiver Experiences of Care Coordination for Recently Discharged Patients: A Qualitative Metasynthesis. West J Nurs Res. 2020 Aug;42(8):649-659. doi: 10.1177/0193945919880183. - **Carnahan JL, Inger L, Rawl SM, Iloabuchi TC, Clark DO, Callahan CM, Torke AM.** Complex Transitions from Skilled Nursing Facility to Home: Patient and Caregiver Perspectives. J Gen Intern Med. 2021 May;36(5):1189-1196. doi: 10.1007/s11606-020-06332-w - **Sawan MJ, Jeon YH, Bond C, Hilmer SN, Chen TF, Wennekers D, Gnjidic D.** Caregivers' experiences of medication management advice for people living with dementia at discharge. J Eval Clin Pract. 2021 Dec;27(6):1252-1261. doi: 10.1111/jep.13551. Epub 2021 Feb 15. PMID: 33586327. |
| **15. IF** Carers have an active involvement in the multi-disciplinary team  **THEN** There is a reduced likelihood of medication errors  **BECAUSE** The relevant knowledge is effectively communicated between carers and HCPs and vice versa | - **Applebaum AJ, Sannes TS.** The importance of honoring family caregiver burden: challenges in mental health care delivery. J Clin Psychol Med Settings. 2025 Jun;32(2):193–201. doi: 10.1007/s10880-024-10051-3. PMID: 39397232. - **Demers C, Patterson C, Archer N, Coallier J, Strachan P, Keshavjee K, Thabane L, Spencer F, Cockhill C, Foster G, Gwadry‑Sridhar F.**  A simple multi‑component intervention improves self‑management in heart failure. Can J Cardiol. 2014;30(10 Suppl):Abstract S0806. doi:10.1016/j.cjca.2014.07.328 - **Parsons C, Canning A.** Experiences of carers and family members when administering medications to people with advanced dementia: an analysis of an online chat forum. Int J Pharm Pract. 2018;26(S1):54-55. doi:10.1111/ijpp.12443 - **Sawan MJ, Jeon YH, Bond C, Hilmer SN, Chen TF, Wennekers D, Gnjidic D.** Caregivers' experiences of medication management advice for people living with dementia at discharge. J Eval Clin Pract. 2021 Dec;27(6):1252-1261. doi: 10.1111/jep.13551. Epub 2021 Feb 15. PMID: 33586327. |
| **16. IF** Patients believe that getting help with medications undermines their independence  **THEN** They may resist offers of help from the carer  **BECAUSE** They may feel ashamed that they are losing autonomy over their health | - **Bruce R, Murdoch W, Kable A, Palazzi K, Hullick C, Pond D, Oldmeadow C, Searles A, Fullerton A, Fraser S, Ling R, Attia J.** Evaluation of Carer Strain and Carer Coping with Medications for People with Dementia after Discharge: Results from the SMS Dementia Study. Healthcare (Basel). 2020 Jul 31;8(3):248. doi: 10.3390/healthcare8030248. - **Chase JD, Russell D, Kaplan DB, Bueno MV, Khiewchaum R, Feldman PH.** "Doing the Right Thing": Family Caregivers Managing Medical and Nursing Tasks in the Postacute Home Health Care Setting. J Appl Gerontol. 2021 Dec;40(12):1786-1795. doi: 10.1177/0733464820961259. - **O’Conor R, Oladejo D, Filec S, Russell A, Lindquist L, Wolf M.** Managing medications across the Alzheimer’s disease spectrum from a patient-caregiver perspective. J Am Geriatr Soc. 2023;71(Suppl 1):S1-S398. doi:10.1111/jgs.18336 - **Song Y, Chen S, Roseman J, Scigliano E, Redd WH, Stadler G.** It takes a team to make it through: the role of social support for survival and self‑care after allogeneic **hematopoietic stem cell transplant. Front Psychol. 2021;12:624906. doi:10.3389/fpsyg.2021.624906** |
| **17. IF** Carers do not feel appreciated by the patient  **THEN** They are likely to be upset  **BECAUSE** They feel taken for granted or undervalued | - **Pereira F, Bieri M, Del Rio Carral M, Martins MM, Verloo H.** Collaborative medication management for older adults after hospital discharge: a qualitative descriptive study. BMC Nurs. 2022 Oct 24;21(1):284. doi: 10.1186/s12912-022-01061-3. |
| **Theme 3: Access to appropriate materials, resources, and support information** | |
| **18. IF** Important information about medications is not given by HCPs to the carer  **THEN** This may lead to medication errors  **BECAUSE** The carer remains unaware of what to do, when to seek help, and who to seek help from | - **Callister C, Jones J, Schroeder S, Breathett K, Dollar B, Sanghvi UJ, Harnke B, Lum HD, Jones CD.** Caregiver Experiences of Care Coordination for Recently Discharged Patients: A Qualitative Meta synthesis. West J Nurs Res. 2020 Aug;42(8):649-659. doi: 10.1177/0193945919880183. - **White CL, Brady TL, Saucedo LL, Motz D, Sharp J, Birnbaum LA.** Towards a better understanding of readmissions after stroke: partnering with stroke survivors and caregivers. J Clin Nurs. 2015 Apr;24(7-8):1091-100. doi: 10.1111/jocn.12739. |
| **19. IF** Carers are given information that is too technical  **THEN** They struggle to carry out medication management  **BECAUSE** They do not understand their tasks | - **Bristol AA, Elmore CE, Weiss ME, Barry LA, Iacob E, Johnson EP, Wallace AS.** Mixed-methods study examining family carers' perceptions of the relationship between intrahospital transitions and patient readiness for discharge. BMJ Qual Saf. 2023 Aug;32(8):447-456. doi: 10.1136/bmjqs-2022-015120. - **Sawan MJ, Jeon YH, Bond C, Hilmer SN, Chen TF, Wennekers D, Gnjidic D.** Caregivers' experiences of medication management advice for people living with dementia at discharge. J Eval Clin Pract. 2021 Dec;27(6):1252-1261. doi: 10.1111/jep.13551. Epub 2021 Feb 15. PMID: 33586327. |
| **20. IF** Carers are only given verbal information  **THEN** They may struggle to remember it, which may lead to adverse events  **BECAUSE** Verbal information is easy to forget | - **Dolu İ, Naharcı Mİ, Logan PA, Paal P, Vaismoradi M.** A qualitative study of older patients’ and family caregivers’ perspectives of transitional care from hospital to home. Res Theory Nurs Pract. 2021;():. doi:10.1891/RTNP-D-20-00067 |
| **21. IF** Carers who have access to physical medication resources (e.g., medications lists)  **THEN** This minimises challenges in remembering information relating to medications  **BECAUSE** They can be reminded by the resource which they can refer back to | - **Barber S, Thakkar K, Marvin V, Franklin BD, Bell D.** Evaluation of My Medication Passport: a patient-completed aide-memoire designed by patients, for patients, to help towards medicines optimisation. BMJ Open. 2014 Aug 19;4(8):e005608. doi: 10.1136/bmjopen-2014-005608. - **Sawan MJ, Jeon YH, Bond C, Hilmer SN, Chen TF, Wennekers D, Gnjidic D.** Caregivers' experiences of medication management advice for people living with dementia at discharge. J Eval Clin Pract. 2021 Dec;27(6):1252-1261. doi: 10.1111/jep.13551. Epub 2021 Feb 15. PMID: 33586327. |
| **22. IF** Carers received tailored medications related information  **THEN** There is a lower risk of medications harm at home  **BECAUSE** They have the relevant knowledge and information needed to confidently deal with specific challenges they face linked to medications | - **Ashcraft S, Wilson SE, Nyström KV, Dusenbury W, Wira CR, Burrus TM;** on behalf of the American Heart Association Council on Cardiovascular and Stroke Nursing and the Stroke Cou**ncil. Care of the patient with acute ischemic stroke (prehospital and acute phase of care): update to the 2009 comprehensive nursing care scientific statement: a scientific statement from the American Heart Association. Stroke. 2021;52(5):e164‑e178. doi:10.1161/STR.0000000000000356** - **Blair J, Volpe M, Aggarwal B.** Challenges, needs, and experiences of recently hospitalized cardiac patients and their informal caregivers. J Cardiovasc Nurs. 2014 Jan-Feb;29(1):29-37. doi: 10.1097/JCN.0b013e3182784123. PMID: 23416934; PMCID: PMC3726572. - **Dolu İ, Naharcı Mİ, Logan PA, Paal P, Vaismoradi M.** A qualitative study of older patients’ and family caregivers’ perspectives of transitional care from hospital to home. Res Theory Nurs Pract. 2021;():. doi:10.1891/RTNP-D-20-00067 - **Goodman H.** Discharging patients from acute care hospitals. Nurs Stand. 2016 Feb 10;30(24):49-57; quiz 60. doi: 10.7748/ns.30.24.49.s47. PMID: 26860177. - **Hahn-Goldberg S, Jeffs L, Troup A, Kubba R, Okrainec K.** "We are doing it together"; The integral role of caregivers in a patients' transition home from the medicine unit. PLoS One. 2018 May 24;13(5):e0197831. doi: 10.1371/journal.pone.0197831. - **Sawan MJ, Jeon YH, Bond C, Hilmer SN, Chen TF, Wennekers D, Gnjidic D.** Caregivers' experiences of medication management advice for people living with dementia at discharge. J Eval Clin Pract. 2021 Dec;27(6):1252-1261. doi: 10.1111/jep.13551. Epub 2021 Feb 15. PMID: 33586327. |
| **23. IF** There is a lack of clarity for patients and carers about medication management, especially for complex regimens  **THEN** Carers will create their own strategies for administration, possibly leading to medication errors  **BECAUSE** Carers will do what they think is the best for the person they are caring for to ensure medication administration continues | - **Carnahan JL, Inger L, Rawl SM, Iloabuchi TC, Clark DO, Callahan CM, Torke AM.** Complex Transitions from Skilled Nursing Facility to Home: Patient and Caregiver Perspectives. J Gen Intern Med. 2021 May;36(5):1189-1196. doi: 10.1007/s11606-020-06332-w - **Parsons C, Canning A.** Experiences of carers and family members when administering medications to people with advanced dementia: an analysis of an online chat forum. Int J Pharm Pract. 2018;26(S1):54-55. doi:10.1111/ijpp.12443 - **Sawan MJ, Jeon YH, Bond C, Hilmer SN, Chen TF, Wennekers D, Gnjidic D.** Caregivers' experiences of medication management advice for people living with dementia at discharge. J Eval Clin Pract. 2021 Dec;27(6):1252-1261. doi: 10.1111/jep.13551. Epub 2021 Feb 15. PMID: 33586327. |
| **24. IF** The discharge materials provided to carers have been co-produced  **THEN** They are better able to effectively carry out home care  **BECAUSE** The materials are relevant and useful to them | - **Carnahan JL, Inger L, Rawl SM, Iloabuchi TC, Clark DO, Callahan CM, Torke AM.** Complex Transitions from Skilled Nursing Facility to Home: Patient and Caregiver Perspectives. J Gen Intern Med. 2021 May;36(5):1189-1196. doi: 10.1007/s11606-020-06332-w - **Fuller TE, Pong DD, Piniella N, Pardo M, Bessa N, Yoon C, Boxer RB, Schnipper JL, Dalal AK.** Interactive Digital Health Tools to Engage Patients and Caregivers in Discharge Preparation: Implementation Study. J Med Internet Res. 2020 Apr 28;22(4):e15573. doi: 10.2196/15573. |
| **25. IF** Carers need help organising medications  **THEN** Access to dose administration aids may be beneficial for appropriate medication use  **BECAUSE** They have clarity on how to manage and administer medications | - **Bruce R, Murdoch W, Kable A, Palazzi K, Hullick C, Pond D, Oldmeadow C, Searles A, Fullerton A, Fraser S, Ling R, Attia J.** Evaluation of Carer Strain and Carer Coping with Medications for People with Dementia after Discharge: Results from the SMS Dementia Study. Healthcare (Basel). 2020 Jul 31;8(3):248. doi: 10.3390/healthcare8030248. - **Sawan MJ, Jeon YH, Bond C, Hilmer SN, Chen TF, Wennekers D, Gnjidic D.** Caregivers' experiences of medication management advice for people living with dementia at discharge. J Eval Clin Pract. 2021 Dec;27(6):1252-1261. doi: 10.1111/jep.13551. Epub 2021 Feb 15. PMID: 33586327. |
| **26. IF** Carers receive proactive help with the knowledge of medications they need to effectively carry out care processes  **THEN** Medical emergencies and adverse events relating to medications can be reduced  **BECAUSE** Carers are better equipped with relevant knowledge and skills to care | - **Andrade LM, Costa MFM, Caetano JÁ, Soares E, Beserra EP.** A problemática do cuidador familiar do portador de acidente vascular cerebral. Rev Esc Enferm USP. 2009;43(1):37-43. doi:10.1590/S0080-62342009000100005 - **Fyfe MM, Alfonso C, Zawatski A, Sikes M.** Blood and marrow transplant caregivers: identifying educational needs. Biol Blood Marrow Transplant. 2017;23(3):S382. doi:10.1016/j.bbmt.2016.12.596 |
| **27. IF** Carers demonstrate their understanding of medications and administration to HCPs before discharge  **THEN** HCPs can identify areas of improvement or errors in the carer's method of care  **BECAUSE** HCPs have an appreciation of the challenges the carer is facing and there is an opportunity to reiterate missing information or correct misunderstanding | - **Cornetta K, Nyariki S, Manji I, et al.** Telehospice for cancer patients discharged from a tertiary care hospital in Western Kenya: a feasibility study. J Pain Symptom Manage. 2023 May;65(5):378–387. doi:10.1016/j.jpainsymman.2023.01.027 - **Horstman MJ, Salas E.** Annals for Hospitalists Inpatient Notes - Caregiver Engagement in Hospital Medicine-The Next Frontier. Ann Intern Med. 2019 Jan 15;170(2):HO2-HO3. doi: 10.7326/M18-3614. PMID: 30641571. - **Schad RF, Lucarotti RL.** Patient-teaching program for home intravenous antimicrobial therapy. Am J Hosp Pharm. 1986;43(2):372-375. doi:10.1093/ajhp/43.2.372 |
| **28. IF** Carers are provided with convenient and timely ways of accessing support (e.g. electronic pillboxes, and phone applications)  **THEN** Medication management is likely to improve  **BECAUSE** They have easier ways to access the knowledge and support needed to deal with challenges they may encounter | - **Ganapathy D, Acharya C, Lachar J, Patidar K, Sterling RK, White MB, Ignudo C, Bommidi S, DeSoto J, Thacker LR, Matherly S, Shaw J, Siddiqui MS, Puri P, Sanyal AJ, Luketic V, Lee H, Stravitz RT, Bajaj JS.** The patient buddy app can potentially prevent hepatic encephalopathy-related readmissions. Liver Int. 2017 Dec;37(12):1843-1851. doi: 10.1111/liv.13494. - **Shannon EM, Mueller SK, Schnipper JL.** Patient, caregiver, and clinician experience with a technologically enabled pillbox: a qualitative study. ACI Open. 2023;7(2):e61–e70. doi:10.1055/s-0043-1775970 - **Wingham J, Frost J, Britten N, Jolly K, Greaves C, Abraham C, Dalal H;** REACH-HF research investigators. Needs of caregivers in heart failure management: A qualitative study. Chronic Illn. 2015 Dec;11(4):304-19. doi: 10.1177/1742395315574765. |
| **29. IF** Carers experience a medication issue that they have not had specific advice on how to manage  **THEN** They will likely come up with their own ‘solution’ e.g., hoard/stop/change delivery of medications, which could be dangerous  **BECAUSE** They feel obligated to find a solution and this is based on their personal knowledge rather than support from a HCP | - **Bull MJ, McShane RE.** Seeking what's best during the transition to adult day health services. Qual Health Res. 2008 May;18(5):597-605. doi: 10.1177/1049732308315174. Epub 2008 Mar 5. PMID: 18322266. - **Parsons C, Canning A.** Experiences of carers and family members when administering medications to people with advanced dementia: an analysis of an online chat forum. Int J Pharm Pract. 2018;26(S1):54-55. doi:10.1111/ijpp.12443 - **Tomlinson J, Silcock J, Smith H, Karban K, Fylan B.** Post-discharge medicines management: the experiences, perceptions and roles of older people and their family carers. Health Expect. 2020 Dec;23(6):1603-1613. doi: 10.1111/hex.13145. Epub 2020 Oct 16. PMID: 33063445; PMCID: PMC7752204. |
| **30. IF** Carers receive adequate guidance and coaching from HCPs  **THEN** They are more confident in their ability to carry out their role  **BECAUSE** They feel more prepared | - **Blair J, Volpe M, Aggarwal B.** Challenges, needs, and experiences of recently hospitalized cardiac patients and their informal caregivers. J Cardiovasc Nurs. 2014 Jan-Feb;29(1):29-37. doi: 10.1097/JCN.0b013e3182784123. PMID: 23416934; PMCID: PMC3726572. - **Bodenheimer T, Berry-Millett R.** Care management of patients with complex health care needs. Synthesis Project Research Synthesis Report No. 19. Robert Wood Johnson Foundation; December 2009. - **Mosca L, Mochari-Greenberger H, Aggarwal B, Liao M, Suero-Tejeda N, Comellas M, Rehm L, Umann TM, Mehran R.** Patterns of caregiving among patients hospitalized with cardiovascular disease. J Cardiovasc Nurs. 2011 Jul-Aug;26(4):305-11. doi: 10.1097/JCN.0b013e3181f34bb3. PMID: 21330929; PMCID: PMC3230071. - **Sawan MJ, Jeon YH, Bond C, Hilmer SN, Chen TF, Wennekers D, Gnjidic D.** Caregivers' experiences of medication management advice for people living with dementia at discharge. J Eval Clin Pract. 2021 Dec;27(6):1252-1261. doi: 10.1111/jep.13551. Epub 2021 Feb 15. PMID: 33586327. |
| **3.2 Receiving validation from HCPs on self-care** | |
| **31. IF** Carers are informed and supported by HCPs about the importance of caring for themselves  **THEN** they feel less guilty for taking care of themselves  **BECAUSE** they feel this is a legitimate activity | - **Andrade LM, Costa MFM, Caetano JÁ, Soares E, Beserra EP.** A problemática do cuidador familiar do portador de acidente vascular cerebral. Rev Esc Enferm USP. 2009;43(1):37-43. doi:10.1590/S0080-62342009000100005 - **Applebaum AJ, Sannes TS.** The importance of honoring family caregiver burden: challenges in mental health care delivery. J Clin Psychol Med Settings. 2025 Jun;32(2):193–201. doi: 10.1007/s10880-024-10051-3. PMID: 39397232. - **Weaver FM, Perloff L, Waters T.** Patients' and caregivers' transition from hospital to home: needs and recommendations. Home Health Care Serv Q. 1999;17(3):27-48. doi:10.1300/J027v17n03_03 |
