# Supplementary material for: Realist review of informal carer involvement in the transition of medicines-related care for patients moving from hospital to home
Source: BMJ Open. 2025 Nov 13;15(11):e107826. doi: 10.1136/bmjopen-2025-107826 (PMC12625932; doi:10.1136/bmjopen-2025-107826)
Supplement: Supplementary file 2 [file bmjopen-2025-107826.reviewer_comments.pdf]

PEER REVIEW HISTORY

BMJ Open publishes all reviews undertaken for accepted manuscripts. Reviewers are asked to complete a checklist review form and are provided with free text boxes to elaborate on their assessment. These free text comments are reproduced below.

ARTICLE DETAILS

Title (Provisional)

A realist review of informal carer involvement in the transition of medicines-related care for patients moving from hospital to home

Authors

Richardson, Charlotte; Atkinson, Olivia; Cooper, Matthew; Black, David; Cooper, Christina; Lindsey, Laura; Nazar, Hamde; Wong, Geoff; Hughes, Carmel

---

VERSION 1 - REVIEW

**Reviewer**	**1**
**Name**	**Powell, Catherine**
**Affiliation**	**University of Bradford Faculty of Health Studies, Centre for Applied Dementia Studies**
**Date**	**21-Aug-2025**
**COI**	**None**

I believe the topic is of importance to carers, patients and professionals. It is good to see involvement of a diverse patient public involvement and engagement group involved through the process, and inclusion of non-English articles.

There are some grammatical errors throughout such as present tense ‘identify’ in the abstract. Please review grammar throughout the manuscript.

The study objective could be clarified into one core objective rather than two parts.

Page 4 line 76- ‘useful’ can give the impression that carers are something like a tool to be used. Is it not that we know carers are involved, and need support/partnership in doing so, or similar?

The definition of medicines management itself needs to be clearer. There is some information provided on page 6 lines 117-119 that could be elaborated on further.

In the supplementary material- CMOCs and illustrative examples of contributing data source(s) please address the following points:

Error in point 6 - ‘with in’

Point 12 define – ‘SDM’

Point 14 - ‘14. IF HCPs communication with patients and carers discuss, negotiates and addresses (where possible) their needs, wants, goals, and preferences’ needs rephrasing

Point 20 – Were there no positive aspects to verbal information found in the literature? For example across diverse groups?

Point 28 - What exactly are the convenient and timely ways of accessing support-compliance aids only? And do they improve all medicines management or just a part of it.

**Reviewer**	**2**
**Name**	**Sawan, Mouna**
**Affiliation**	**The University of Sydney**
**Date**	**02-Sep-2025**
**COI**	**None**

Thank you for the opportunity to review this important and well-written paper. The study offers valuable insights into reducing medication-related harm in older adults following hospital discharge by improving guidance on medication management provided to carers. I offer the following comments to enhance the reporting of the realist review:

Abstract

I commend the authors for incorporating public involvement in the realist review. Could you please specify: The number of patient/public members involved in the focus groups? The number of focus groups conducted? How these focus groups contributed to the development of CMOCs and the program theory?

Introduction

Please clarify the age group of patients referred to in the first sentence: “The transition of ‘older adult patients’ from hospital to home…”

Similarly, please specify the age group of carers included in the review.

In paragraph two, please include a reference to support the statement: Patients transitioning from hospital to home can experience significant disruption to their usual routines, especially when medicines are changed.

Please add polypharmacy and the use of high-risk medications as additional factors contributing to increased MRH during and after hospital discharge.

Methods

Please provide a definition of medication management that guided the review methodology.

Please elaborate on the coding process: Who conducted the coding? How were codes grouped into categories? Were these categories reviewed by the authors to reach consensus?

Regarding the inclusion criteria: The review focuses on the transition from hospital to home. Does 'home' refer exclusively to community-dwelling individuals, or were nursing homes/long-term care settings also included? Please clarify the post-discharge setting in both the methods and introduction.

Further detail is needed on the patient and public involvement and engagement group:

How many participants were involved? How were meetings conducted? What was the structure of the interview guide? How was data collected? How did their involvement influence the findings? Please specify the carer organisations that participated.

For Step 4: Synthesising evidence and drawing conclusions, please explain how realist logic was applied during data analysis to develop CMOCs, and how consensus among authors was achieved.

Results

Can you please report on how focus groups with the public involvement group contributed to the development of CMOCs and the program theory?

Regarding Theme 3: In addition to the need for written information, did any studies highlight the importance of alternative modes of information delivery (e.g., online resources, videos, apps)?

VERSION 1 - AUTHOR RESPONSE

Reviewer 1

I believe the topic is of importance to carers, patients and professionals. It is good to see involvement of a diverse patient public involvement and engagement group involved through the process, and inclusion of non-English articles.

Thank you for your positive feedback. We are pleased you recognise the value of public contributors and of non-English articles.

1. There are some grammatical errors throughout such as present tense 'identify' in the abstract. Please review grammar throughout the manuscript.
   Grammar has been reviewed throughout the manuscript and in the example of 'identify' in the abstract this has been corrected.
2. The study objective could be clarified into one core objective rather than two parts.
   Thank you for your suggestion. We appreciate that the aim is somewhat unusual in its language and presentation. After further consideration, we have chosen not to change this given it is in keeping with usual convention for realist work and also it reflects the published protocol for this work.
3. Page 4 line 76- 'useful' can give the impression that carers are something like a tool to be used. Is it not that we know carers are involved, and need support/partnership in doing so, or similar?
   We agree that the use of the term 'useful' here did not fully capture the message we were

trying to convey. As this text was placed under the heading 'What is already known on the subject' and this is not part of BMJ Open's required format, the Editor has asked that the statement be removed which we have done.

4. The definition of medicines management itself needs to be clearer. There is some information provided on page 6 lines 117-119 that could be elaborated on further.
   We have added some further information relating to medicines management and have included a clear definition within the section suggested.
5. In the supplementary material- CMOCs and illustrative examples of contributing data source(s) please address the following points:
   a. Error in point 6 - 'with in' corrected
   b. Point 12 define – 'SDM' corrected
   c. Point 14 - '14. IF HCPs communication with patients and carers discuss, negotiates and addresses (where possible) their needs, wants, goals, and preferences'
      In relation to Point 14, this has been corrected to: 'IF HCP's communication with patients and carers discusses, negotiates and addresses (where possible) their needs, wants, goals, and preferences', this has also been corrected in figure 4.
   d. Point 20 – Were there no positive aspects to verbal information found in the literature? For example, across diverse groups?
      Our findings did not specifically identify positive aspects relating to verbal information but we imagine there are some advantages reported elsewhere in the literature. Rather the findings were related to when only verbal methods are used to deliver information where patients may struggle to retain all relevant information. Hence, a combination of verbal plus written is probably a better option for retention of information.
   e. Point 28 - What exactly are the convenient and timely ways of accessing support- compliance aids only? And do they improve all medicines management or just a part of it.
      This is an interesting point and requires careful consideration. The data has demonstrated that 'convenient and timely' will mean different things to different people and this can be identified and managed through much of what is captured in 'Theme 2: Understanding the carers' priorities, role and responsibilities through shared decision making'. It is also therefore likely that this will contribute to medicines management in different ways for different people. The example contributing papers within the supplementary materials provided include a range of support options to illustrate this can be wide ranging.

Reviewer 2

Thank you for the opportunity to review this important and well-written paper. The study offers valuable insights into reducing medication-related harm in older adults following hospital discharge by improving guidance on medication management provided to carers. I offer the following comments to enhance the reporting of the realist review:

Thank you for your review which has helped us further strengthen the paper as follows.

Abstract

1. I commend the authors for incorporating public involvement in the realist review. Could you please specify: The number of patient/public members involved in the focus groups? The number of focus groups conducted? How these focus groups contributed to the development of CMOCs and the program theory?
   Thank you for your positive feedback on the PPIE within the project. We agree the detail on numbers would be useful as well as more clarity as to what the contributions actually were. We have now added this additional detail.

Introduction

2. Please clarify the age group of patients referred to in the first sentence: "The transition of 'older adult patients' from hospital to home..."
   The paper which referred to support in this sentence does not focus on a particular age group or type of patient. Rather the authors concluded that patients who are older, frail or have complex medical conditions or medicines are most vulnerable. We have added this detail into the paper.
3. Similarly, please specify the age group of carers included in the review.
   We have clarified within the introduction that the focus was only on adult carers. This information was also already provided within Table 1: Eligibility criteria.
4. In paragraph two, please include a reference to support the statement: Patients transitioning from hospital to home can experience significant disruption to their usual routines, especially when medicines are changed.
   A reference has now been added to support this statement.
5. Please add polypharmacy and the use of high-risk medications as additional factors contributing to increased MRH during and after hospital discharge.
   As per point 2 above, this information has been added in terms of describing the patients most at risk of safety errors during the transition from hospital to home.

Methods

6. Please provide a definition of medication management that guided the review methodology.
   This was also commented on by the other reviewer and as such we have added this within the Introduction where medicines management is first noted.
7. Please elaborate on the coding process: Who conducted the coding? How were codes grouped into categories? Were these categories reviewed by the authors to reach consensus?
   We have further elaborated on the coding process in the text.
8. Regarding the inclusion criteria: The review focuses on the transition from hospital to home. Does 'home' refer exclusively to community-dwelling individuals, or were nursing homes/long-term care settings also included? Please clarify the post-discharge setting in both the methods and introduction.
   Throughout our work, 'home' related to community-dwelling patients and data relating to care homes were excluded. This was reported in Table 1 but we have added further detail here and in the Introduction.
9. Further detail is needed on the patient and public involvement and engagement group: How many participants were involved? How were meetings conducted? What was the structure of the interview guide? How was data collected? How did their involvement influence the findings? Please specify the carer organisations that participated.
   Within the PPIE section we have attempted to include all of the detail requested to better explain the role and influence of the group. Meetings were conducted online and each started with a presentation by a member of the research team followed by guided discussion. The presentation content varied depending on the stage of the research but typically included an update on the work and the latest iteration of CMOCs/PT. As this was part of PPIE and not a focus group for data collection, feedback was verbal or written *via* the 'chat' function with a facilitator taking notes. Members were encouraged to provide positive and negative feedback, and make suggestions for improvements based on their experience as a carer. This feedback helped to confirm and refute CMOCs particularly relating to the specific language used within them. We have added an example of one of the carer groups.

10. For Step 4: Synthesising evidence and drawing conclusions, please explain how realist logic was applied during data analysis to develop CMOCs, and how consensus among authors was achieved.
    Due to the word count for this paper, we have chosen not to provide extensive detail on this stage of the method, especially as it is already published within our protocol paper. We have more clearly referenced this paper within the stage 4 section and we have also added detail on how consensus was achieved.

Results

11. Can you please report on how focus groups with the public involvement group contributed to the development of CMOCs and the program theory?
    As per your earlier comments about the Abstract this has now been added to the main text within the Methods subheading 'Patient and public involvement and engagement contribution'.
12. Regarding Theme 3: In addition to the need for written information, did any studies highlight the importance of alternative modes of information delivery (e.g., online resources, videos, apps)?
    This is an important point and with regards to CMOC-22 this is specifically about the need for written information (e.g., as opposed to verbal) but does not specify mode of delivery. Therefore the CMOC would still apply for written information via an app or a website. Videos were not specifically identified in relation to this part of our study.
